# Neural correlates of eye contact in face-to-face verbal interaction: An EEG-based study of the extraversion personality trait

**DOI:** 10.1371/journal.pone.0219839

**Published:** 2019-07-25

**Authors:** Nur Syahirah Roslan, Lila Iznita Izhar, Ibrahima Faye, Hafeez Ullah Amin, Mohamad Naufal Mohamad Saad, Subarna Sivapalan, Samsul Ariffin Abdul Karim, Mohammad Abdul Rahman

**Affiliations:** 1 Centre for Intelligent Signal & Imaging Research (CISIR), Universiti Teknologi PETRONAS, Perak, Malaysia; 2 Department of Electrical & Electronics Engineering, Universiti Teknologi PETRONAS, Perak, Malaysia; 3 Department of Fundamental & Applied Sciences, Universiti Teknologi PETRONAS, Perak, Malaysia; 4 Centre for Excellence in Teaching & Learning (CETaL), Universiti Teknologi PETRONAS, Perak, Malaysia; 5 Centre for Smart Grid Energy Research (CSMER), Universiti Teknologi PETRONAS, Perak, Malaysia; 6 Faculty of Medicine, University of Kuala Lumpur Royal College of Medicine Perak, Perak, Malaysia; University of California Irvine, UNITED STATES

## Abstract

The extraversion personality trait has a positive correlation with social interaction. In neuroimaging studies, investigations on extraversion in face-to-face verbal interactions are still scarce. This study presents an electroencephalography (EEG)-based investigation of the extraversion personality trait in relation to eye contact during face-to-face interactions, as this is a vital signal in social interactions. A sample of healthy male participants were selected (consisting of sixteen more extraverted and sixteen less extraverted individuals) and evaluated with the Eysenck's Personality Inventory (EPI) and Big Five Inventory (BFI) tools. EEG alpha oscillations in the occipital region were measured to investigate extraversion personality trait correlates of eye contact during a face-to-face interaction task and an eyes-open condition. The results revealed that the extraversion personality trait has a significant positive correlation with EEG alpha coherence in the occipital region, presumably due to its relationship with eye contact during the interaction task. Furthermore, the decrease in EEG alpha power during the interaction task compared to the eyes-open condition was found to be greater in the less extraverted participants; however, no significant difference was observed between the less and more extraverted participants. Overall, these findings encourage further research towards the understanding of neural mechanism correlates of the extraversion personality trait—particularly in social interaction.

## Introduction

In the Big Five theory, the extraversion personality trait is one of the five major traits alleged to form human personalities [1, 2]. People who are high in extraversion can be characterised as talkative, gregarious, energetic, and assertive and vice versa for those with low extraversion. This personality trait is believed to influence an individual's preferences and performance in various areas, such as work and education [3–5]. For instance, more extraverted persons are the most likely to seek professions with a higher level of task significance, power, and feedback [3]. They love to be assigned the responsibility to create transformation, as well as handle multiple functions [3]. This is believed to be due to their preference for feeling a sense of significance, and for evaluating their own performance in relation to that of others.

Furthermore, more extraverted persons prefer to engage with people and enjoy social interaction, while less extraverted persons tend to be quiet, reserved and less sociable [1, 6]. After social activities, more extraverted persons achieve significant increases in positive affectivity as compared to less extraverted individuals [7]. These differences influence more and less extraverted persons to behave differently when interacting with others, face-to-face. For instance, compared to more extraverted persons, less extraverted persons were found to have larger general inhibitions, which led them to hesitation in sharing their ideas in a group discussion [4]. Presumably, because of this general inhibition, less extraverted persons prefer to communicate and interact via the internet, while more extraverted persons prefer face-to-face social interaction [8, 9].

As we know, during a face-to-face social interaction, eye contact is an important social signal [10]. This can be described as two persons gazing at each other's eyes [11, 12]. These gazing activities could be a cue of one person's attention towards another [13–15]. For instance, three-month-old infants' smiling behaviour declined when an interacting person gazed away after having made eye contact [16]. In terms of the extraversion personality trait, [17] summarized several studies and found that more and less extraverted persons have differences in terms of gaze direction, duration, and frequency. They reported that the direction, duration, and frequency of gaze were found to be toward, long, and often, respectively, in more extraverted persons; and corresponded to avoidance, short, and seldom in less extraverted persons [17]. Supporting these findings was a study that claimed the extraversion personality trait is directly proportional to the attention an individual commits to the eyes of another person during social tasks [18]. By referring to these findings, we could say that more and less extraverted persons could be differentiated based on eyes contact during social events. Although many studies showed differentiation of extraversion personality trait in terms of eye contact, research involving electroencephalography (EEG) remains scarce—particularly involving the naturalistic paradigm of face-to-face verbal interaction [19, 20].

EEG is an effective neuroimaging modality to study individual differences in personality [21–23]. This is because of its direct measurement of neural signals and remarkable temporal resolution compared to other neuroimaging modalities, such as functional magnetic resonance imaging (fMRI) and positron emission tomography (PET) [24]. One of the well-known theories of extraversion was proposed by Eysenck [25], which stated that the extraversion personality trait has a negative correlation with cortical arousal. Based on this theory, some EEG studies discovered that more extraverted persons demonstrated lower levels of cortical arousal and less extraverted persons demonstrated higher levels of cortical arousal, as measured through EEG alpha oscillations [26, 27]. Additionally, several researchers investigated the extraversion personality trait and EEG alpha oscillations in posterior regions, such as the parietal and occipital regions [28–31]. Although these studies generally showed a positive association between the extraversion personality trait and EEG alpha oscillations in the posterior region [28, 30], one study was unable to find such a significant association [29]. Along with technological development in removing eye movement artefacts in the frontal region, [32] investigated positive and negative empathic moods with the extraversion personality trait in broad brain regions, including frontal (F3, F4), temporal (T3, T4), and occipital (O1, O2). They claimed that extraversion was related to larger alpha amplitude in widespread regions. Furthermore, [26] focused on the frontal region (Fp1, Fp2, F3, F4, F7, F8, Fz) in an investigation of EEG alpha oscillations, while participants opened and closed their eyes on instruction. They suggested similar findings to those of [32] in that more extraverted persons exhibited a larger alpha amplitude compared to that of less extraverted persons. Investigations of EEG alpha oscillations and the extraversion personality trait are ongoing in various aspects, such as intelligence [33], memory performance [34, 35], creativity and originality [27], and emotional processing [36]. For analysis, several studies focused on the entire brain including the occipital region [27, 36], while others excluded the occipital region [34, 35]. Such differences in the brain regions of interest in these studies could partly be due to the different sets of objectives and experimental tasks considered.

Based on our review, although there have been many studies investigating EEG correlates of the extraversion personality trait, none have incorporated the aspect of eye contact during a face-to-face interaction. Hence, the aim of this study was to investigate EEG correlates of extraversion in relation to eye contact by measuring EEG alpha coherence and power in the occipital region during a face-to-face interaction task. EEG alpha oscillations were considered on the basis of Eysenck's well-known theory of extraversion [25], and for having been explored in many EEG studies related to extraversion personality trait [26, 27, 37].

## Methods

### Participants

Ninety-one healthy male students of Universiti Teknologi PETRONAS volunteered to participate in the experiment. The volunteers were exclusively male so as to avoid any possible gender effect on the EEG results [38, 39]. According to their self-report, they were right-handed, had normal or corrected to normal vision, had no hearing impairment, were not suffering from or having family history related to cognitive disorder, were not taking any drugs or any medication, nor experiencing chronic mental stress or adverse psychology states. All volunteers had to complete two personality tests—the Eysenck Personality Inventory (EPI) and Big Five Inventory (BFI)—to determine their level of extraversion before they could participate in this study [40, 41]. The volunteers were characterised as less extraverted if they scored less than 50% for both EPI and BFI, and more extraverted if they scored more than 50% for both EPI and BFI. After a screening session, 50 volunteers were excluded due to conflicting results between their personality tests. Of the 41 remaining volunteers who met the requirement, only 32 returned to the study, consisting of 16 less and 16 more extraverted participants. These were between 18 and 23 years old (M = 19.53 and SD = 1.22). All participants gave informed consent and were paid for their participation in the EEG experiment for this study.

This research was approved by the Medical Research Ethics Committee of the University of Kuala Lumpur Royal College of Medicine Perak, Malaysia.

### Experimental task: A face-to-face interaction task

**Fig 1:**
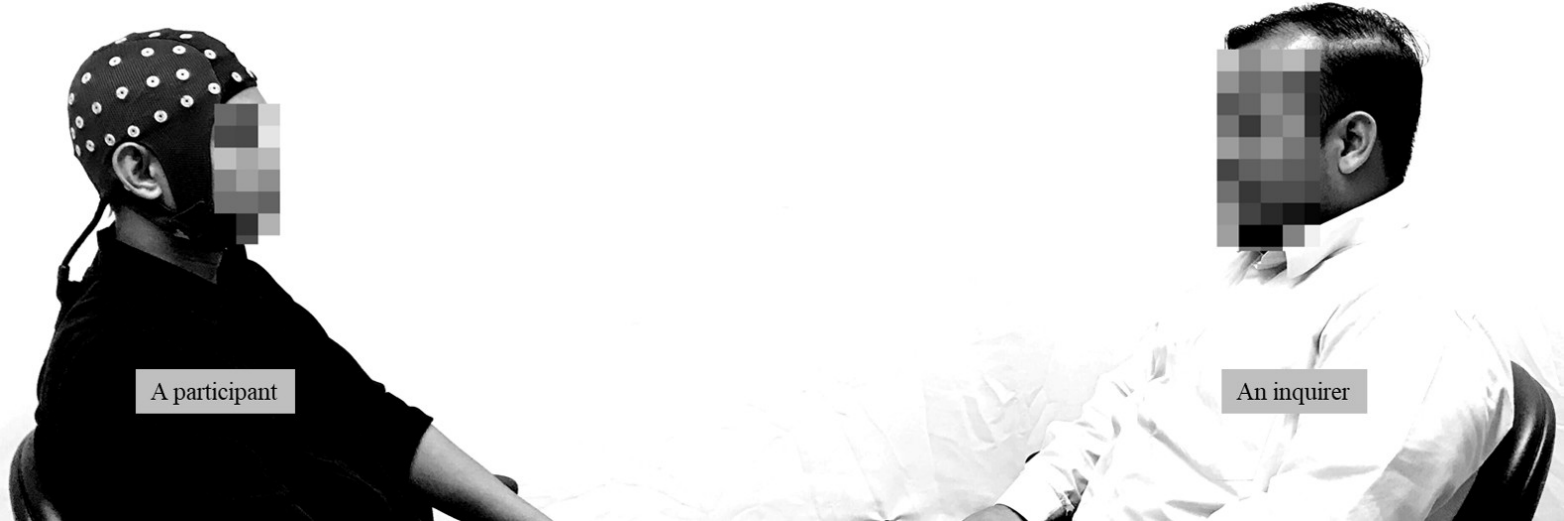
Face-to-face interaction task: A participant wearing an EEG cap is seated facing an inquirer. (The individual in this manuscript has given written informed consent—as outlined in the PLOS consent form—to publish these case details.)

**Fig 2:**
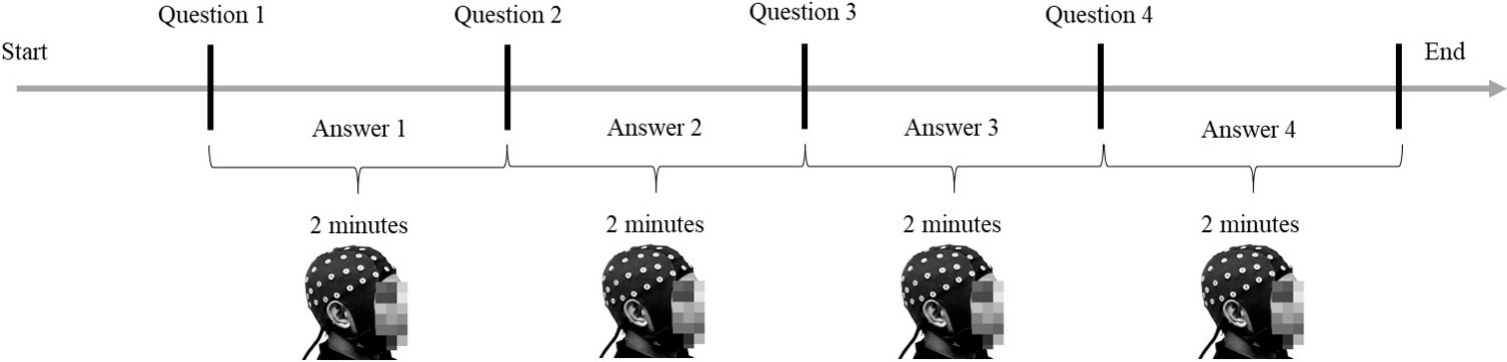
Timeline of the face-to-face interaction task. (The individual in this manuscript has given written informed consent—as outlined in the PLOS consent form—to publish these case details.)

The experiment conducted involved a form of a face-to-face interaction task, which was performed individually by each participant. During the task, the participant was instructed to sit facing an inquirer who was a stranger to them (see Fig 1). This face-to-face interaction task with a stranger was selected for its potential to induce stress and anxiety related to social awkwardness, which could be one of the challenges for less extraverted participants. In the task, four questions were asked by the inquirer to the participants, one at a time. The participants were instructed to answer each question spontaneously, within two minutes. The instructor stopped the session when the time had reached two minutes. See Fig 2 for a detailed timeline of the interaction task. All questions remained unknown to the participants until the inquirer asked them.

### Procedure

Prior to the beginning of the experiment, participants were provided with information about the experiment and their informed consents were obtained. The enrolled participants were then seated in a partially sound-attenuated EEG experiment room to perform the experiment individually. Subsequently, the EEG device was set up, which included assisting participants in donning an EEG cap. EEG recordings began with a 5-minute eyes-closed period, followed by a 5-minute eyes-open period. During the eyes-open period, participants were advised to focus on one point and avoid eye movement. Next, a practice session was held in which they needed to answer one question within two minutes. The purpose of this practice session was to ensure participants provided maximal EEG data quality during the actual experimental task. During the practice session, participants were guided on how to minimise EEG artefacts, such as placing their hands on their laps to avoid hand movements and thereby improve the quality of the EEG data. Such artefacts are due to the fact that EEG devices are sensitive and easily interrupted by other unwanted electrical activities. After the practice session, participants needed to introduce themselves. They were then asked to perform the face-to-face interaction task. For each question in the interaction task, the EEG recording started when the inquirer began to ask the question and stopped when the time to answer reached two minutes (see Fig 2). Following the interaction task, participants were asked to fill a self-assessment survey related to the task (see Table 1). At the end of their session, each participant was instructed to keep all questions asked during the interaction task strictly confidential. This was to avoid other participants from preparing their answers before the experiment.

### EEG acquisition

The EEG data was recorded using a 32-channels eego^TM^ sports system (ANT Neuro, Netherlands) with Ag/AgCl scalp electrodes arranged based on the International 10–10 System. The CPz electrode was selected as a reference electrode. The impedance of each electrode was kept below 10 kΩ. The EEG data were digitised at a sampling rate of 512 Hz.

### Data analysis

#### EEG preprocessing

The recorded EEG data were pre-processed using BESA Research 6.0 (www.besa.de). First, the raw EEG data were bandpass filtered with a cut-off frequency of 0.53–48 Hz. Eye artefacts (movements and blinks) and muscle movements were corrected using the Berg & Scherg method [42] implemented in the BESA software as used by [43]. The cleaned EEG data were then exported to MATLAB for EEG features extraction.

#### EEG features

**EEG alpha coherence**: This is one of the methods used to measure brain functional connectivity by measuring signals from two electrodes, or two brain regions. In mathematics, coherence can be defined as the ratio of normalised cross-power spectrum to auto-power spectrum [44]. Coherence ranges from 0 to 1, where 0 means the corresponding two signals are not functionally coherent and 1 means the two signals are fully functionally coherent with a constant phase difference, suggesting that the two electrodes (brain areas) are working together. EEG coherence can be calculated using Eq (1) [44]:

$$C^{2}{}_{xy}\left(f\right)=\frac{{\left|P_{xy}\left(f\right)\right|}^{2}}{P_{xx}\left(f\right)P_{yy}\left(f\right)}$$ (1)

where *f* is the frequency, *P*_*xy*_ is the cross-power spectrum between the signals of two electrodes *x* and *y*, *P*_*xx*_ is the auto-power spectrum for the signals of electrode *x*, and *P*_*yy*_ is the auto-power spectrum for the signals of electrode y.

For EEG coherence in this study, only 19 of the 32 electrodes were selected based on the 10–20 placement system. The number of pairs was calculated using pairs $=\;(\frac{n(n-1)}{2})$, where *n* = 19 resulted in 171 pairs. The EEG coherence for all 171 pairs was calculated for the four oscillations (alpha, delta, theta, and beta) using Eq (1). The EEG alpha coherence for the paired occipital electrodes (O1 and O2) were extracted and averaged within the participants and within the questions to investigate extraversion personality trait correlates of eye contact during the interaction task.

**EEG alpha power**: In this study, EEG alpha power was measured in the eyes-open condition to serve as a baseline, and during an interaction task. A fast Fourier transform (FFT) with a Hanning window was used on 512 samples with a 50% overlap between successive 2-second segments (1024 points) for estimating the power of the EEG signals. The EEG alpha power during the interaction task and in the eyes-open condition in the occipital region (electrodes O1 and O2) was extracted and averaged at a standard frequency of 8–13 Hz. Subsequently, the EEG alpha power data were averaged within the participants and within the interaction task questions. The mean EEG alpha power for the interaction task was compared with that of the eyes-open baseline. Increase or decrease in the EEG features (e.g. EEG alpha power) during the experimental task in comparison with the baseline were computed for each participant by [45]:

$$A_{EB}=\left(S_{E}-S_{B}\right)/S_{B}$$ (2)

Where *A*_*EB*_ is the increase/decrease in the EEG alpha power (positive/negative values) during the experimental task as compared to that of the eyes-open baseline, *S*_*E*_ is the EEG alpha power during the experimental task, and *S*_*B*_ is the EEG alpha power in the eyes-open condition.

#### Statistical analysis

As mentioned, two personality tests (i.e. the EPI and BFI) were used for personality assessment. The extraversion scores of all participants were computed by converting the EPI and BFI scores to percentages and averaging them. Pearson correlation was used to determine the correlation between the averaged extraversion score (E-score) and both personality tests. The E-score was used throughout the analysis.

Subsequently, the mean ± standard deviation of the EEG features and self-assessment survey items of two possible groups—more and less extraverted—were computed. The significant results of these two possible groups were measured using one-way analysis of variance (ANOVA). Additionally, ANOVA was used to identify the optimal EEG features and self-assessment survey items of the extraversion personality trait. These optimal features and items were selected based on the highest *F*-value of the ANOVA.

Finally, the main correlation of extraversion personality with the optimal EEG feature and self-assessment survey item were explored using the linear regression method. Two linear regression scatterplots were analysed to demonstrate the possible correlation. For all statistical analyses, the Statistical Package for the Social Sciences (SPSS) software platform was used, in which *p*-values smaller than 0.05 indicate statistically significant data. The data were represented in mean ± standard deviation, unless otherwise specified.

## Results

In this section, we discuss the results of all tested data in this study. First, we discuss the results of the two personality tests, including the proposed E-score. Second, we discuss the results of the EEG features—EEG alpha coherence and EEG alpha power—in relation to the extraversion personality trait. Third, we discuss the results of the self-assessment survey related to the face-to-face interaction task. The statistical tests mentioned in the ‘Statistical analysis’ section were used to determine the significance of all data representing extraversion.

### Personality tests

In this study, two personality tests—the EPI and BFI—were used to evaluate the level of extraversion in each participant. The EPI and BFI are self-report questionnaires where the EPI contained 57 “YES” or “NO” items and the BFI contained 44 5-point scale items (1 = disagree strongly…5 = agree strongly). Of the 57 items in the EPI, 24 were used to assess the extraversion personality trait, while for BFI, 8 out of 44 items were used for the same. As stated in the ‘Statistical analysis’ section, the averaged E-score of each participant was computed by averaging the scores of their EPI and BFI after converting them to percentages. More extraverted participants scored more than 50%, while less extraverted participants scored less than 50%. We proposed to employ the E-score as there was consistency between the extraversion facet scales of the EPI and BFI [46]. Moreover, the Pearson correlation revealed that the EPI was significantly correlated with the BFI (*r* = 0.896, *p* < 0.001). The mean E-scores for the more and less extraverted participants were found to be 68.18 ± 8.80 and 35.22 ± 7.60, respectively. For validation of the proposed E-score, the Pearson correlation showed that it was significantly correlated with both the EPI (*r* = 0.982, *p* < 0.001) and BFI (*r* = 0.964, *p* < 0.001). These results indicated that the E-score could be used to represent each participant's level of extraversion in the statistical analysis [47].

### EEG features

**Fig 3:**
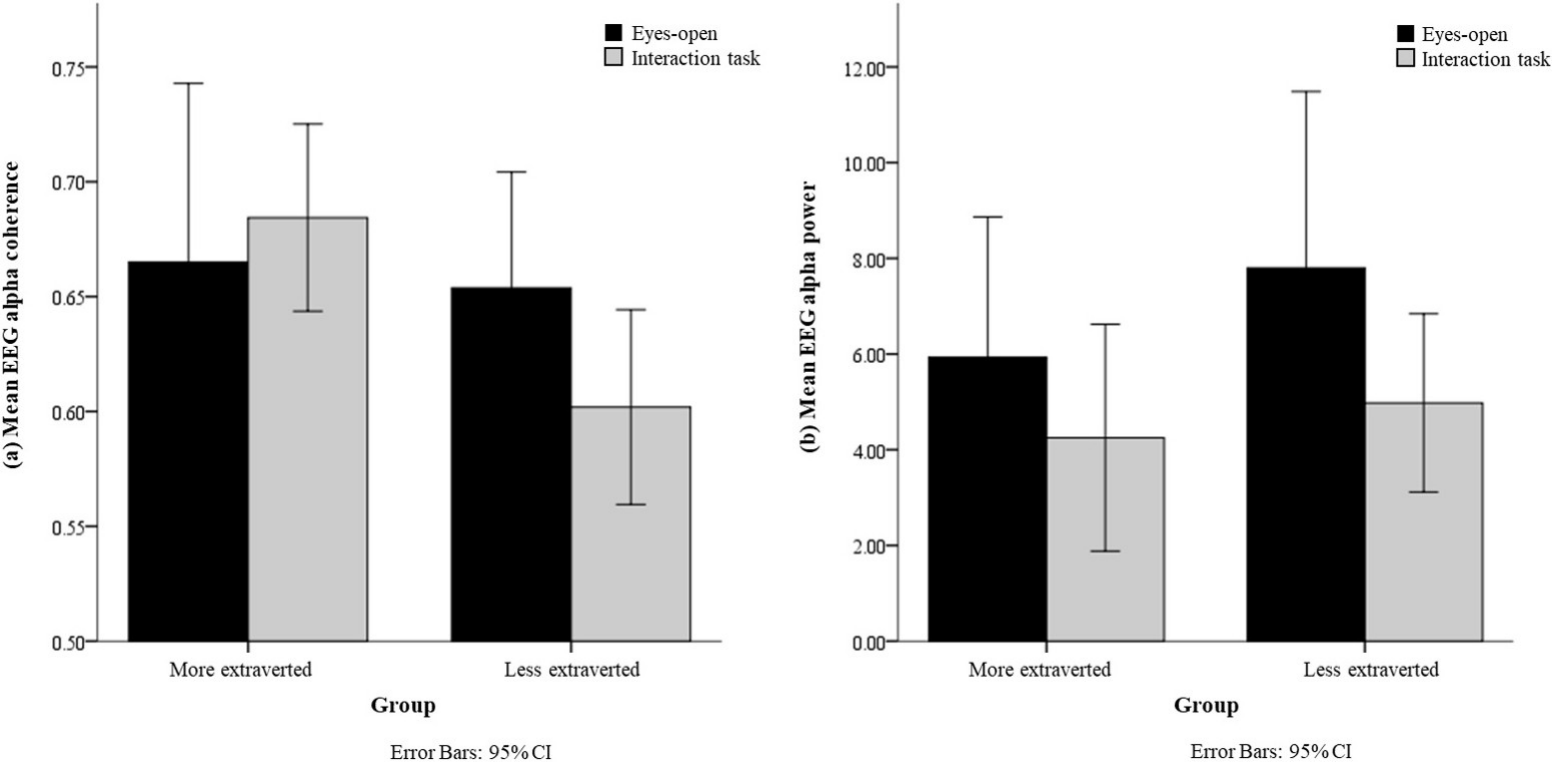
(a) Mean EEG alpha coherence and (b) mean EEG alpha power in the occipital region in the eyes-open condition and face-to-face interaction task.

Possible group differences of the extraversion personality trait—with respect to two EEG features (i.e. EEG alpha coherence and EEG alpha power) in the occipital region during the face-to-face interaction task and in the eyes-open condition—were analysed. The mean EEG alpha coherence in the occipital region in the eyes-open condition for both groups were similar (more extraverted = 0.66 ± 0.15; less extraverted = 0.65 ± 0.09), as illustrated in Fig 3A. For the mean EEG alpha power in the eyes-open condition, the less extraverted participants exhibited higher levels (7.80 ± 6.91) than the more extraverted participants (5.93 ± 5.51), as illustrated in Fig 3B. However, as can be seen in Table 1, the differences between the group means of both EEG features in the eyes-open condition were not statistically significant (*p* > 0.05). Thus, for these cases, null and alternative hypotheses cannot be rejected and accepted, respectively, for the EEG features in the eyes-open condition.

For the face-to-face interaction task, the mean EEG alpha coherence of the more extraverted participants (0.68 ± 0.08) was higher than that of the less extraverted participants (0.60 ± 0.08), as illustrated in Fig 3A. Additionally, the difference between the group means of EEG alpha coherence was statistically significant (see *p*-value in Table 1). Thus, null and alternative hypotheses could be rejected and accepted, respectively, for EEG alpha coherence during the interaction task. For the mean EEG alpha power during the interaction task, the less extraverted participants exhibited higher EEG alpha power levels (4.98 ± 3.50) than the more extraverted participants (4.25 ± 4.45), as illustrated in Fig 3B. However, the difference between the group means of EEG alpha power during the interaction task was not statistically significant (*p* > 0.05; see Table 1).

**Table 1:** One-way ANOVA of EEG features and self-assessment survey.

		*F*	*p-value*	$\eta_{p}^{2}$	Observed power^(ii)^
**EEG alpha coherence**	**Eyes-open**	0.067	0.798	0.002	0.057
	**Interaction task**	8.934	0.006^(i)^	0.229	0.824
**EEG alpha power**	**Eyes-open**	0.716	0.404	0.023	0.130
	**Interaction task**	0.263	0.612	0.009	0.079
**(a) I was comfortable sitting in front of strangers.**		20.589	0.000^(i)^	0.407	0.992
**(b) I was comfortable sharing my answer or idea during the group discussion.**		12.273	0.001^(i)^	0.290	0.924
**(c) I voiced my ideas or opinion spontaneously.**		4.892	0.035^(i)^	0.140	0.572
**(d) I would prefer to give feedback verbally.**		7.418	0.011^(i)^	0.198	0.75

Note: (a)-(d) items in self-assessment survey;

(i) *p-value* < 0.05;

(ii) computed using alpha = 0.05

Additionally, the differences between the EEG features during the interaction task and eyes-open condition were calculated. The results revealed that the mean EEG alpha coherence of the more extraverted participants was increased (0.10 ± 0.40) during the interaction task as compared to that of the eyes-open condition. In comparison, the mean EEG alpha coherence of the less extraverted participants (-0.07 ± 0.11) was decreased during the interaction task compared to the eyes-open condition. Although there was dissimilarity in the mean EEG alpha coherence between the more and less extraverted participants, there was no statistically significant difference between them: *F*(1,30) = 2.678, *p* > 0.05. The same lack of significance was found for differences between the mean EEG alpha power of the more (-0.09 ± 1.05) and less extraverted (-0.16 ± 0.56) participants: *F*(1,30) = 0.052, *p* > 0.05.

**Fig 4:**
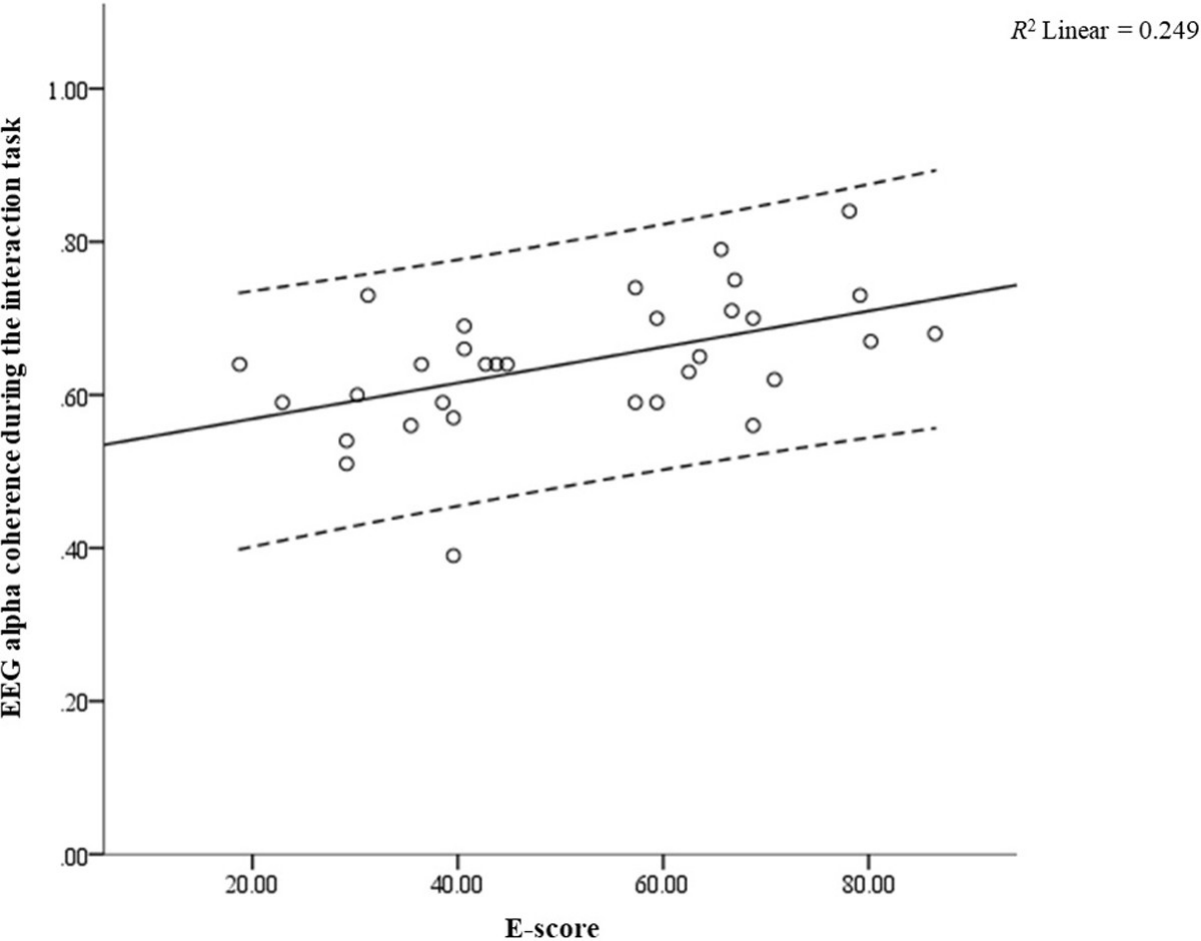
Linear regression relation between extraversion personality trait (E-score) and EEG alpha coherence during face-to-face interaction task, (*F*(1,30) = 9.946, *p* = 0.004).

Additionally, ANOVA was then used to identify the optimal EEG features. Based on the results of the ANOVA for each of the EEG features (see Table 1), EEG alpha coherence during the interaction task demonstrated the highest *F*-value (*F* = 8.934). After obtaining the optimal EEG feature, a linear regression was employed to determine the influence of the extraversion personality trait on EEG alpha coherence during the interaction task. To evaluate the linearity, a scatterplot was made of EEG alpha coherence during the interaction task against the extraversion personality trait (E-score), and a superimposed regression line was plotted. As illustrated in Fig 4, the linear regression established that the extraversion personality trait could be used to predict the EEG alpha coherence during the interaction task with statistical significance: *F*(1,30) = 9.946, *p* < 0.05, with *R*^*2*^ = 0.249; a large effect size according to Cohen [48]. The regression equation was: predicted EEG alpha coherence during the interaction task = 0.52 + 0.002x (E-score).

### Self-assessment survey

As stated in the ‘Procedure’ section, after performing the face-to-face interaction task, each participant needed to fill in a self-assessment survey related to the task. All items in the self-assessment were rated on a 5-point Likert scale ranging from 1 (strongly disagree) to 5 (strongly agree). This self-assessment had an acceptable internal consistency, as determined by a Cronbach's alpha of 0.76 [49]. The results of the survey showed that the more extraverted participants reported that they felt more comfortable sitting in front of a stranger and sharing ideas, were more spontaneous in voicing those ideas, and had a greater preference for giving feedback verbally compared to the less extraverted participants (see Table 2). The difference between the group means for all items in the self-assessment survey were found to be statistically significant (*p* < 0.05), as presented in Table 1.

**Table 2:** Descriptive statistics for self-assessment survey by more and less extraverted participants.

	More extraverted		Less extraverted	
	M	SD	M	SD
**(a) I was comfortable sitting in front of strangers.**	4	0.73	2.81	0.75
**(b) I was comfortable sharing my answer or idea during the group discussion.**	4.25	0.58	3.50	0.63
**(c) I voiced my ideas or opinion spontaneously.**	4.31	0.87	3.63	0.89
**(d) I would prefer to give feedback verbally.**	3.69	0.95	2.75	1.00

Note: M = mean; SD = standard deviation.

**Fig 5:**
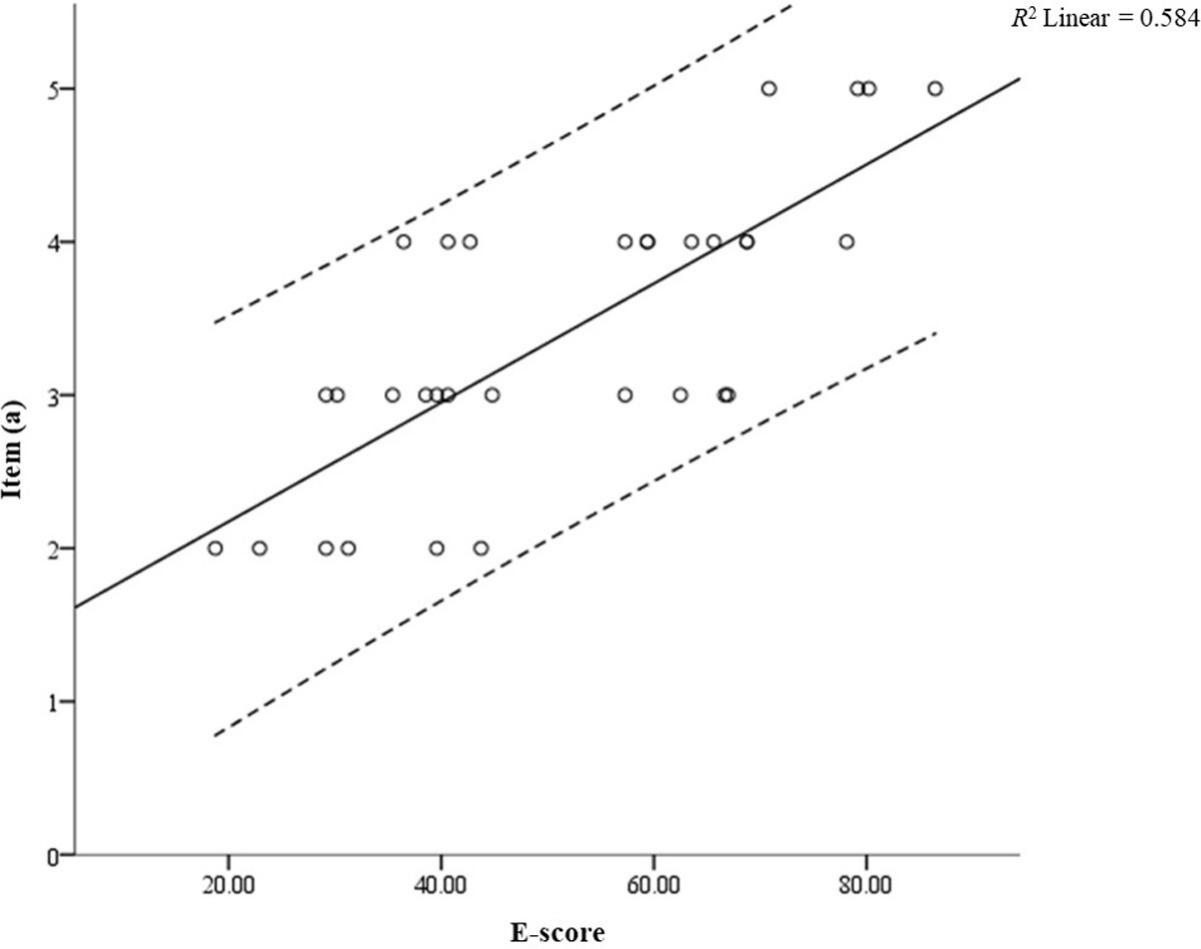
Linear regression relation between extraversion personality trait (E-score) and item (a): comfortableness while sitting in front of a stranger; (*F*(1,30) = 42.044, *p* < 0.0001).

As mentioned in the ‘Statistical analysis’ section, ANOVA was used to identify the optimal self-assessment survey item by referring to the highest *F*-value of all items. Based on the ANOVA results in Table 1, item (a) demonstrated the highest *F*-value (*F* = 20.589). After obtaining the optimal self-assessment survey item, a linear regression was employed to determine the correlation between the extraversion personality trait and the comfortableness of the participants sitting in front of the strangers (item (a)). To evaluate the linearity, a scatterplot was made of item (a) against the extraversion personality trait (E-score), and a superimposed regression line was plotted. As illustrated in Fig 5, the linear regression established that the extraversion personality trait could be used to predict the item (a) with statistical significance: *F*(1,30) = 42.044, *p* < 0.0001, with *R*^*2*^ = 0.584; a large effect size according to Cohen [48]. The regression equation was: predicted item (a) = 1.40 + 0.04x (E-score).

## Discussion

The aim of this study was to investigate correlates between neural signals in a specific brain region and the extraversion personality trait. Specifically, the investigation focused on an EEG-based study of extraversion correlates with eye contact during a face-to-face interaction task with a stranger. We mainly focused on eye contact as it is a crucial signal during social interactions. We also analysed the consistency of the EEG features with a self-assessment survey of the interaction task. The findings of this study regarding the EEG features and self-assessment survey are believed to have provided a reliable outcome in the investigation of the extraversion personality trait during the face-to-face interaction task.

The present study found that the mean EEG alpha coherence (i.e. the connectivity between brain regions) during the face-to-face interaction task was the optimal EEG feature for extraversion. This result suggests that the mean EEG alpha coherence during the interaction task could expose extraversion differences better than EEG features in the eyes-open condition (baseline/resting state). Our findings support that the EEG resting state cannot be used to recognise the extraversion personality trait [50]. During the interaction task, the mean EEG alpha coherence in the occipital region of the more extraverted participants was significantly higher than that of the less extraverted participants. The EEG alpha coherence results indicate a stronger connectivity between the left (O1) and right (O2) occipital regions in the more extraverted participants during the interaction task. As the occipital region involves visual processing [51], we deduced that this high connectivity in the occipital region occurred in the more extraverted participants as a result of their consistent eye contact during the interaction task. This presumption is in line with studies that described the extraversion personality trait as being directly proportional to the attention or eye contact one commits to the eyes of another [18, 52]. In further support of this, a linear regression in this study demonstrated a significant positive correlation between the extraversion personality trait and mean EEG alpha coherence in the occipital region during the interaction task (see Fig 4). Therefore, we believe the more extraverted participants had more consistent eye contact with others than the less extraverted participants.

Moreover, the interpretation of the EEG alpha coherence is also supported by behavioural data. The linear regression demonstrated a significant positive correlation between the extraversion personality trait and the comfort of the participants sitting in front of a stranger (see Fig 5). This comfortableness is believed to enable the more extraverted participants to maintain better eye contact than the less extraverted participants [53, 54]. Due to this comfortableness, the mean EEG alpha coherence of more extraverted participants was increased during the interaction task. Besides an increased level of comfort, the more extraverted participants were also found to be more comfortable and spontaneous in sharing their ideas than the less extraverted participants. They also reported a preference for giving feedback verbally. All results from the self-assessment survey were predictable, as it is well known that more extraverted individuals are more outgoing and sociable, while less extraverted individuals are the opposite [1, 6, 55, 56]. These results provided further evidence that more extraverted individuals are easily adapted to response as compared to less extraverted individuals [57, 58]. Furthermore, the results support the notion that more extraverted individuals build up their characteristic of low arousal from response organisation, while less extraverted individuals' characteristic of high arousal means they tend to derive inhibition from response organisation and prefer stimulus analysis [58]. In this case, the interaction task was believed to cause the more extraverted participants to be more comfortable than the less extraverted participants.

Besides mean EEG alpha coherence, we also observed a decrease in mean EEG alpha power between the more extraverted and less extraverted participants during the interaction task compared to the baseline. As illustrated in Fig 3A, the decrease in EEG alpha power during the interaction task compared to the eyes-open condition was greater in the less extraverted participants than the more extraverted participants. Based on our review, there is inconsistency in the reasoning for this decrease in EEG alpha oscillations. Some studies claim that a decrease in EEG alpha power in the occipital region is due to the attention of an individual on the location of a visual target [51, 59]. Another study claims that the decrease is due to a wandering mind [60]. Similarly, we were unable to reach a solid conclusion regarding the relationship between the extraversion personality trait and EEG alpha power during the interaction task. This likely contributed to difficulty in achieving statistically significant results for EEG alpha power correlates of the extraversion personality trait during the face-to-face interaction. In comparison, the EEG alpha coherence provided more substantial evidence representing the extraversion personality trait. Presumably, this is because coherence is measured by considering the signals from two electrodes, while power was measured from a single electrode [44]. Additional research remains necessary to clarify the relationship between EEG alpha power in the occipital region and the extraversion personality trait during the interaction task.

There are certain limitations to this study which deserve further investigation. First, the sample was limited to right-handed male students aged 18–23 years old. Therefore, the assumptions in this study could not be used to generalise about female, non-students, left-handed or middle-aged adult groups. Second, this study was limited to two groups—i.e. more and less extraverted. As such, multiple comparisons could not be made. Third, this study employed a cross-sectional design. Longitudinal studies are required for further insight into neural mechanism correlates of the extraversion personality trait during the interaction task. For instance, less extraverted participants could repeat the experiment after a few months to determine whether their neural signals have changed. Such longitudinal neuroimaging studies are needed to objectively investigate personality changes throughout the lifespan to support subjective study which claimed personality could be changed—particularly in adolescence and old age [61]. Fourth, this study only mentioned the general relation between the extraversion personality trait and eye contact using EEG signals. Further analysis—mainly on the degree of eye contact and extraversion—should be added and thorough measurements should be conducted. Fifth, the self-assessment survey in this study was limited to only four items. We believe adding more items to the survey could assist us in justifying the EEG results. In general, this study was limited to only an EEG investigation and self-assessment survey of the interaction task. Other physiological measurements, such as functional near-infrared spectroscopy (fNIRS), electrocardiography (ECG) or electrodermal response, could assist us in gaining further insight into the extraversion personality trait during the face-to-face interaction task.

In conclusion, this study revealed that the extraversion personality trait was positively correlated with EEG alpha coherence in the occipital region representing neural correlates of eye contact during a face-to-face verbal interaction. The resulting EEG alpha coherence during the interaction task was found to be in agreement with the self-assessment survey collected in this study. This emphasises that an EEG-based investigation related to social interaction can reveal the extraversion personality trait, thus providing an alternative to EEG resting state investigations. Contradictory to those of the EEG alpha coherence, the EEG alpha power findings in this study showed that further investigation is required to clarify inconsistencies in the literature. Overall, the results obtained in this study have laid the foundation upon which to develop our understanding of neural mechanism correlates of the extraversion personality trait in face-to-face interactions or social activities.
